# The Field of Cell Competition Comes of Age: Semantics and Technological Synergy

**DOI:** 10.3389/fcell.2022.891569

**Published:** 2022-05-11

**Authors:** Kieran Maheden, Vivian Weixuan Zhang, Nika Shakiba

**Affiliations:** School of Biomedical Engineering, University of British Columbia, Vancouver, BC, Canada

**Keywords:** synthetic biology, cell competition, development, stem cells, molecular recording

## Abstract

Stem cells experience many selective pressures which shape their cellular populations, potentially pushing them to skew towards dominance of a few break-through clones. An evolutionarily conserved answer to curb these aberrant selective pressures is cell competition, the elimination of a subset of cells by their neighbours in a seemingly homogenous population. Cell competition in mammalian systems is a relatively recent discovery that has now been observed across many tissue systems, such as embryonic, haematopoietic, intestinal, and epithelial compartments. With this rapidly growing field, there is a need to revisit and standardize the terminology used, much of which has been co-opted from evolutionary biology. Further, the implications of cell competition across biological scales in organisms have been difficult to capture. In this review, we make three key points. One, we propose new nomenclature to standardize concepts across dispersed studies of different types of competition, each of which currently use the same terminology to describe different phenomena. Second, we highlight the challenges in capturing information flow across biological scales. Third, we challenge the field to incorporate next generation technologies into the cell competition toolkit to bridge these gaps. As the field of cell competition matures, synergy between cutting edge tools will help elucidate the molecular events which shape cellular growth and death dynamics, allowing a deeper examination of this evolutionarily conserved mechanism at the core of multicellularity.

## Clonality and Competition

The life of a stem cell is that of growth or death, persistence or differentiation. Across generations and within our tissues, these cells find themselves racing towards their limits—space, molecular signals, and the abundance of their progeny relative to peers. These selective pressures inherently define cellular populations, shaping them over the organism’s lifetime depending on the demands of the environment. Heterogeneity is lost over time in several stem cell populations, as a few cells give rise to offspring that outlast and overtake neighbouring clones. Intestinal stem cells, caught in a perpetual rush to out-divide each other, come to grow in abundance and their progeny dominate an environment either by chance or by mutations that provide an edge over their neighbours (Lopez-Garcia et al., 2010; Snippert et al., 2010; Baker et al., 2014; Snippert et al., 2014). As a result, intestinal tissue and the constituent crypts eventually become more homogenous as individual clones out-persist the multicellular population. Interestingly, the reverse phenomenon is seen in epithelial tissues, where clonal diversity increases as cells acquire mutations, giving rise to a birth of clones with divergent family trees (Alcolea et al., 2014; Colom et al., 2021). This ultimately culminates with a single clone “breaking through”, gaining mutations that allow it to overtake its environment. One key difference between these two patterns of cellular competition is the nature of the tissue it occurs within—where one clone may come to outcompete its neighbours in an epithelial layer, spatially separated crypts isolate clones, limiting their spread. While perhaps seeming straightforward, the dynamics of clonal competition and neoplastic growth are increasingly complex, and have been recently well reviewed elsewhere (Marongiu et al., 2021).

Competition between cellular neighbours has been reported in several stem cell populations. Haematopoietic stem cell populations tend towards clonality in an age-dependent manner (Genovese et al., 2014; Jaiswal and Ebert, 2019; Silver et al., 2021). Similarly, a small set of embryonic stem cells establish clonal contributions to reproductive tissue (Kanatsu-Shinohara et al., 2006; Kanatsu-Shinohara et al., 2016; Nguyen and Laird, 2021; Yang et al., 2021) and exhibit skewed contributions to germ layer specification during embryonic development (Park et al., 2021). Despite the clear importance of clonal dynamics in many of our tissues, we lack an understanding of the implications of these differences and how they arise. Often, these changes in clonality are associated with oncogenesis or tissue function detereriation (Suda et al., 2018; Yokoyama et al., 2019), but the impact of tissue-specific clonality or *aclonality* at the organism level remains relatively unexplored. This dynamic, where stochasticity or chance mutations heavily skew a valuable stem cell population, creates a pressure for “cheater” clones to emerge and overtake a bodily niche—often at the expense of organism survival.

One evolutionarily conserved answer to control this selective pressure is a set of cellular interactions dubbed cell competition (CC). CC has traditionally been defined as the elimination of a subset of cells by their neighbours within a seemingly homogenous cell population. This dynamic has been proposed as a conserved quality control mechanism, functioning to select against deleterious mutations (Sancho et al., 2013; Ji et al., 2021). To date, CC has been demonstrated in embryonic (Clavería et al., 2013; Sancho et al., 2013; Ellis et al., 2019), intestinal (Snippert et al., 2014; Suijkerbuijk et al., 2016; Ellis et al., 2019; Flanagan et al., 2021; Scheuer et al., 2021), haematopoietic (Bondar and Medzhitov, 2010), gonadal (Jin et al., 2008; Rhiner et al., 2009; Nguyen and Laird, 2021), and epithelial tissues (Snippert et al., 2014; Suijkerbuijk et al., 2016; Kon et al., 2017; Ellis et al., 2019; Flanagan et al., 2021; Scheuer et al., 2021) scattered throughout several model species. The “why” of cell competition is mostly unknown, however insights from embryonic competition suggest cell function is a determinant of competitive ability (Clavería et al., 2013; Hashimoto and Sasaki, 2019). Indeed, literature on competition has outlined its role in monitoring for epithelial cell polarity (Brumby and Richardson, 2003; Igaki et al., 2009), embryonic tissue size and morphology (Orietti et al., 2021), and in maintaining an organism’s lifespan (Merino et al., 2015).

To date, the field of CC has mostly been concerned with the novelty of “loser” cell removal by neighbouring “winners.” However, how these dynamics impact the residing tissue and its function, or the implications for the host organism are broadly unknown. The field, as it progresses, is then faced with a challenge: designing experiments and formulating hypotheses that encompass the full complexity of multicellular systems at the cellular, tissue, and organism scale. Ultimately, in capturing and understanding the information flow between these scales, we can learn the processes by which these levels shape each other. This is key to connecting the evolutionary basis and molecular mechanisms that orchestrate the competition that shapes multicellular populations (Maheden et al., 2021).

### CLARIFYING COMPETITION

As the field of cell competition has grown quickly, so too has the number of terms used to describe these cellular interactions. With reports of CC becoming commonplace across tissues and organisms, it is timely to revisit and standardize vocabulary across the field. Since the earliest examples of CC were reported, the field has borrowed terminology from evolutionary biology, which has an established and rich history of capturing inter-species competition. However, the broad use of the same terms in evolutionary and developmental biology may be a source of confusion for those new to the field.

The use of the term “fitness” is a clear example of this. In the context of CC, “fitness” is widely used to describe the inherent qualities of winner cells that confer their ability to eliminate losers (Clavería et al., 2013; Sancho et al., 2013; Bowling et al., 2019; Madan et al., 2019; Lima et al., 2021). However, the ability to eliminate losers is often defined by the cell’s “fitness”; an argument that begins where it ends. Indeed, there is no agreed-upon definition for “fitness”, even in the ecological realm (Peacock, 2011); so too, there is a discrepancy in how different experts may interpret the mechanistic underpinnings of cellular “fitness”. An example of this disconnect is seen in the comparison between “fitness” in *Drosophila* epithelial cells as compared to the mammalian haematopoietic system. Cell competition studies using the haematopoietic system describe “fitter” cells as those that exhibit a growth advantage, often conferred by genetic mutations that allow these clones to overtake the niche (Watson et al., 2020). In contrast, cell competition studies involving *Drosophila* describe “fitter” cells as those that are able to eliminate neighbouring cells that are less fit by inducing their apoptosis (Casas-Tinto et al., 2010; De La Cova et al., 2014; Nagata and Igaki, 2018). This demonstrates the disconnect in the use of the term “fitness” across different subfields of competition: the same term is used to describe different phenomena in a context-dependent manner. Indeed, the historical use of the term “fitness” makes it particularly challenging to adapt for the CC field and may warrant careful use as a result.

More specific and nuanced terminology may help the budding CC field avoid inadvertently grouping together distinct biological phenomena and allow for the growing library of CC mechanisms to be catalogued. At the moment, “fitness” is used as a non-specific umbrella term to describe different facets of a cell’s ability to dominate in a given niche. As the field matures, and a more nuanced understanding of the various mechanistic drivers of this phenotype develops, the catch-all term “fitness” can be replaced by more specific terminology.

**FIGURE 1:**
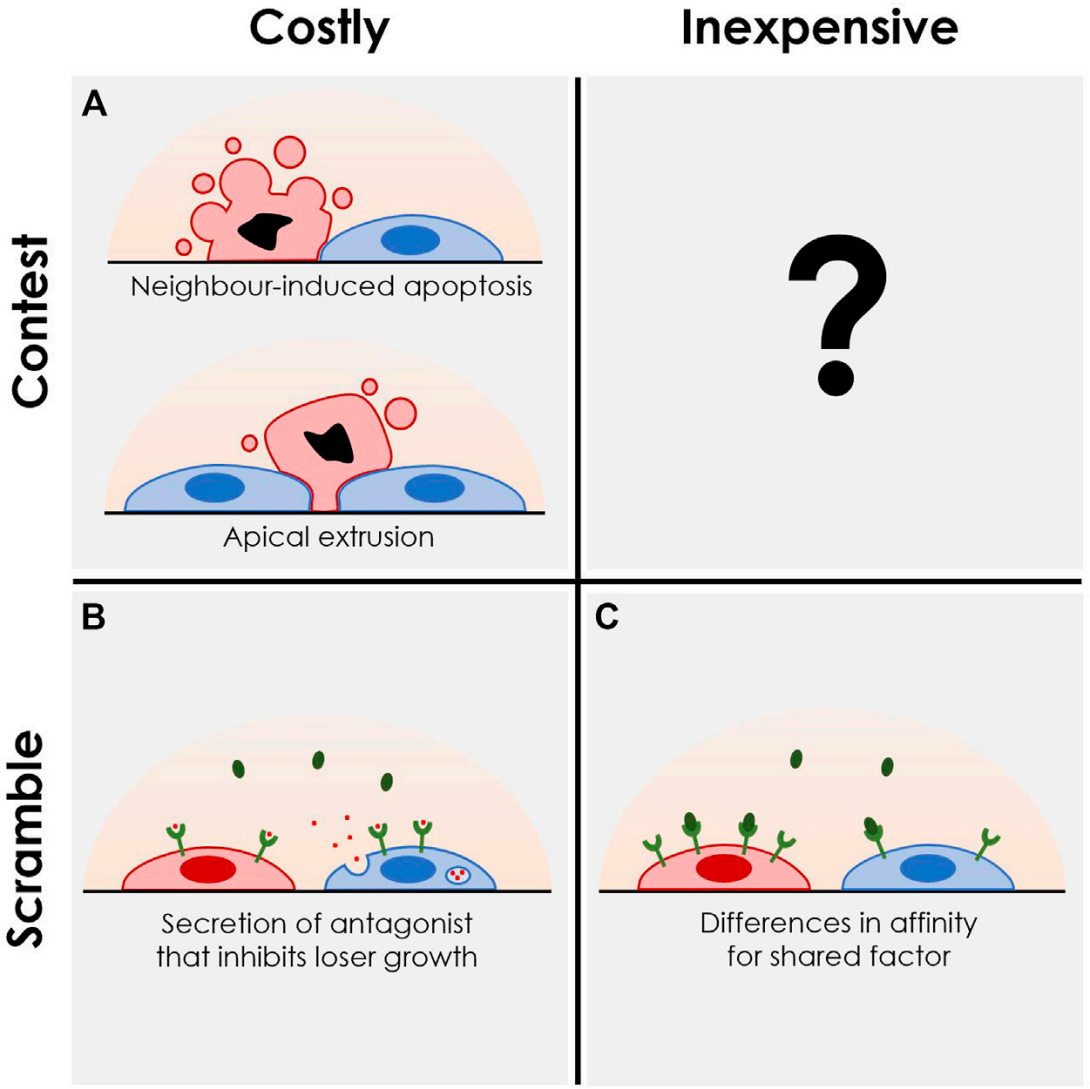
Systematic characterization of the various modes of cell competition. We propose contextualizing competitive interactions *via* two axes: *Costly* versus *inexpensive*; *scramble* versus *contest*. “Costly competition” includes behaviours or molecular interactions that do not yield immediate benefit for the cell or population outside of the elimination of loser cells from that population. “Inexpensive competition” involves less risk as these behaviours or interactions would be expected to always benefit the cell, regardless of environment. “Scramble” involves a rush to accumulate a limited number of resources, whereas “contests” involve direct interactions, comparisons, or sensing between competing cell populations. **(A)** Examples of “costly contests” include direct apoptotic induction of loser cells by winners as seen in mouse embryonic stem cell models (Díaz-Díaz et al., 2017; Bowling et al., 2018b; Lima et al., 2021), or *via* extrusion of losers from the underlying substrate by neighbours as seen in tissue culture models (Hogan et al., 2009; Kajita et al., 2010). **(B)** “Costly scrambles” have been seen in intestinal stem cell models, where *apc*-mutant cells with constitutive WNT signaling secrete a WNT antagonist, NOTUM, crippling WNT signaling in their wildtype neighbours and limiting their ability to persist in the stem cell state (Flanagan et al., 2021; Scheuer et al., 2021). **(C)** “Inexpensive scramble” encompasses a wide range of mechanisms which ultimately result in differences in abundances between two populations by affecting the independent growth or survival of the competitors. This can be driven by different cell cycle rates, death rates, or different required thresholds and affinities for environmental factors, all ultimately resulting in inequality of effective growth rates independent of intercellular interactions. A straightforward example can be seen again within the intestinal stem cell niche, where faster-dividing mutant intestinal stem cells overtake their wildtype neighbours over time (Snippert et al., 2014), or with hematopoietic stem cells having different abilities to persist long term based on growth factor receptor expression (Cosgun et al., 2014; Shin et al., 2014). To date, no clear examples of “inexpensive contest” have been observed.

Similarly, “competition” is used to describe distinct phenomena across fields. We propose to redefine “competition” along two dimensions, each serving to capture distinct biological mechanisms that lead to cellular elimination in multicellular settings: *costly* versus *inexpensive* competition, and cellular *contest* versus *scramble* (Figure 1) (Hibbing et al., 2010). *Costly* competition invovles situations in which a cell or population of cells expend energy to eliminate other competitors, while experiencing no immediate benefit in terms of proliferation or abundance. On the other hand, *inexpensive* competition describes situations where competition is driven by processes that directly increase the abundance of the cells or populations involved. This is seen in situations where one cell population has a cell-autonomous propensity to outgrow or outpersist other populations in a given niche—a property that remains unchanged in the presence of a competitor cell population. *Scramble* refers to competition that affects an extracellular intermediate that drives a change to the cellular microenvironment. By contrast, *contest* refers to a direct action between cells, without involvement of an intermediate resource, such as when cells induce apoptosis in or extrude their neighbours (Sun et al., 2014; Bowling et al., 2018a).

Published reports of CC in various organisms and tissue types can be categorized along these two axes, which together define four quadrants (Figure 1). As an example of a *costly contest*, loser mouse embryonic stem cells undergo apoptosis when mixed with neighbouring winner cells (Figure 1A) (Díaz-Díaz et al., 2017). From the standpoint of these winners, elimination of loser clones requires energy and time and does not yield immediate benefit (i.e., an increase in winner cell numbers) if the loser were not present. An example of a *costly scramble* is shown by Flanagan et al. in which secretion of NOTUM by *apc*-mutant winner cells suppresses wildtype losers’ ability to persist in the niche by targeting their WNT signaling (Figure 1B) (Flanagan et al., 2021). In such a situation, NOTUM production and secretion presents as the cost of eliminating wildtype neighbours, with the ultimate benefit of increased availability of the stem cell niche for these *apc* mutants due to their lack of dependence on WNT signaling. Other examples may involve cells competing by racing for access to a limited nutrient or physical space within a niche. On the other hand, *inexpensive scramble* has been seen in hematopoietic stem cell competition, where clones of stem cells with specific receptor expression levels or mutations predispose them to surviving in that stem cell state over the long term (Figure 1C) (Cosgun et al., 2014; Shin et al., 2014; Bowman et al., 2018; Jaiswal and Ebert, 2019). While an example of an *inexpensive contest* has not been reported to our knowledge, a possible example could involve a contact-mediated elimination of loser cells based on differential expression of surface proteins involved in cell adhesion. In this way, the removal of loser cells from an adherent substrate or cellular niche would be driven by a passive differential adhesion mechanism rather than an active process of extrusion by winners. Ultimately, we hope that this standardization of the vocabulary used by researchers will aid in the discovery and communication of nuanced insights in this burgeoning field.

### CELL COMPETITION ACROSS SCALES

**FIGURE 2:**
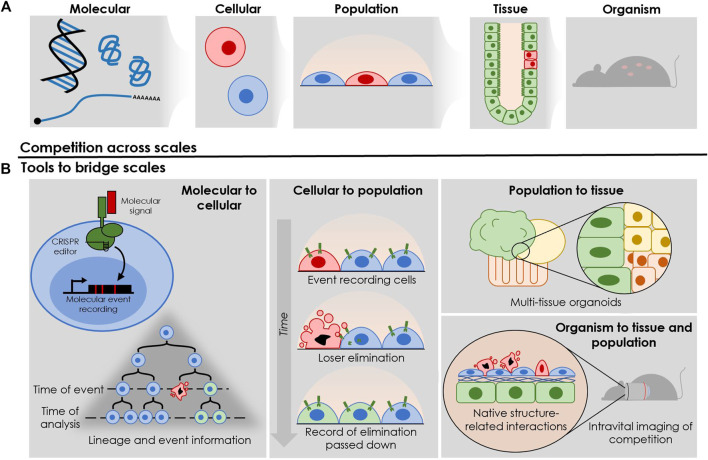
Utilizing recent technological advancements to gain insights into various layers of complexity during cell competition. **(A)** The different biological levels influencing and influenced by competitive interactions between cells. **(B)** Following the biological levels shown in Figure 2A, recent technologies provide unprecedented access to information flow across scales. Molecular level: event recording allows for the tracking of molecular events with single-cell resolution—in this case, the elimination of a loser cell from a mosaic tissue. Cellular level: the surrounding cells can record this elimination, allowing researchers to observe this molecular event in the cellular history through end-point single-cell analysis. Cell population level: complex, multi-organoid systems allow for interplay between tissue types, and for emergent behaviours between cells that typically interact *in vivo* but are traditionally isolated in simple 2-dimentional cultures *in vitro.* Tissue and organism level: intravital imaging enables the investigation of interactions in their *in vivo* context, allowing access to competitive interactions in complex tissue architecture and cellular milieu.

Cell competition is a phenomenon that bridges across scales, where molecular changes at the single cell level drive phenotypic changes within multicellular tissues (Figure 2A). These underlying dynamics ultimately shape the highest biological scale—that of organism viability, which is the level at which evolutionary selection pressures are dealt. Current assays used to investigate CC typically do not span multiple biological scales: molecular, single cell, cell population, tissue, and organism. This leaves a mechanistic disconnect in our understanding of how changes involving CC at one biological level drive outcomes at another. Here, we will review how current assays characterize CC at various scales, outlining gaps in our collective understanding of the biology while highlighting opportunities to push the boundaries of the field through cutting-edge advances in genetic and cellular technologies.

At the molecular level, studies have explored the impact of genetic perturbations on the ability of winner cells to contribute to cellular populations, both *in vitro* and *in vivo*. Claveria et al. and Sancho et al. both show that early embryonic cells measure Myc content relative to their neighbours, and cells with lower Myc levels are eliminated by neighbours with higher Myc (Clavería et al., 2013; Sancho et al., 2013). While these studies reveal the impact of key genetic perturbations in driving the ability of individual cells to overtake populations, they do not capture their impact on higher-level tissue function or structure, and organism outcomes. Of note is that changes to gene expression shown to drive CC need not involve genetic engineering or mutations to the DNA itself. Cells may receive signals from their microenvironment, including cell-cell interactions, that converge on the cellular processor and drive CC behavour by affecting gene expression (Maheden et al., 2021; Shakiba et al., 2021). Similarly, epigenetic changes can also drive CC-relevant gene expression changes. Exciting open questions remain to be answered, including exploring the contributions of inputs to the cellular processor on CC outcomes.

Others have investigated competition at the tissue level. Bondar and Medzhitov demonstrated cell competition in haematopoietic stem and progenitor cells using an *in vivo* bone marrow repopulation assay (Bondar and Medzhitov, 2010). Here, the connection is made between molecular perturbations and population level outcomes, as increased relative p53 expression was shown to confer loser status by marking cells for senescence and extrusion from the niche (Bondar and Medzhitov, 2010). Follow-up studies on the long-term health and blood compartment function of the recipient mice may provide more insight into how winner-loser status at the tissue level shapes survival and viability at the organism level. Indeed, these findings would help to bridge our understanding of the evolutionary role of CC.

Our understanding of the role of CC at the organismal level is relatively underdeveloped. The clearest example comes from work in *Drosophila*, studying the gene *azot*, a gene required for loser cell elimination (Merino et al., 2015). This work outlined several roles for *azot*, showing it is critical for survival post-UV irradiation and maintanence of a normal lifespan through elimination of loser clones arising during both. The connection between competition and lifespan or survivial post-insult has not been made in mammalian systems, and the percise implications of carrying tissues rife with uneliminated losers is an open question.

The phenomenon of CC has captured the attention of scientists with core expertise that span different model systems, technical skills, and cell types. With the exciting interdisciplinary nature of the field comes the challenge of interpreting results that span different biological scales and systems. Mixing assays, involving *in vitro* co-culturing of winner and loser cells on a 2-dimensional surface, have traditionally dominated the field. These assays connect molecular-level genetic perturbations to cell-level events such as apoptosis, killng, and proliferation. Tissue-level studies connect genetic perturbations to the succesful function of the organ, and so on with organismal studies. The disconnect lies in the fact that cell mixing assays provide little insight into the impact of genetic perturbations on any level above that of the cellular. On the other hand, organism-level assays often do not preserve information about underlying genetic perturbations and how they affect cellular and tissue function.

While an understanding of how genetic perturbations drive CC outcomes at the cellular level is powerful for some contexts, such as engineering cells that survive and thrive in cell manufacturing bioprocesses, expanding the scope of our studies to incorporate how these perturbations shape the emergent structure and function of tissues may open the door to the robust derivation of lab-grown tissues for regenerative medicine. Undoubtedly, these insights would also be powerful for our fundamental biological understanding of tissue development and homeostasis as well, with implications for treating disease. Increased cross-disciplinary collaboration would benefit the field by connecting scientists investigating CC from different angles and encouraging broader perspectives.

In mammalian systems, much has recently been learned from pushing beyond *in vitro* mixing studies. During development, it was shown that the same mosaic *epidermis* consisting of a pair of genotypes would show differing mechanisms of elimination, depending on the timing and maturity of the tissue (Ellis et al., 2019). The same work showed that when competition and elimination of one of the genotypes was blocked, tight-junction organization and barrier function was compromised. A recent example leveraging intravital imaging further outlined the critical role of tissue architecture in defining competitive interactions. In skin epithelium, consisting of mosaic hair follicle stem cells (wildtype or with hyperactive WNT/β-catenin signaling), it was shown that mutant cells form growths and protrusions that are encapsulated and stifled by surrounding wildtype cells. This dynamic eventually resulted in a return to homeostatic tissue organization and elimination of mutant cells, a phenomenon only observable when studying the original cellular architecture.

### EXPANDING THE COMPETITION ASSAY TOOLKIT

As the field of CC progresses and the complexity of our questions increase, there are opportunities to engage cutting-edge techniques from the fields of systems and synthetic biology (Figure 2B). Looking to previous work, mathematical modeling has played a critical role in understanding the evolutionary pressures that drive clonal competition in cancer (Gatenby and Vincent, 2003; Vermeulen et al., 2013). Indeed, computational approaches using live-cell imaging data have been successful at disentangling cellular factors involved in 2-dimensional mechanical competition (Gradeci et al., 2019). Additionally, modeling of intestinal stem cell clonality over time initially predicted that the progressive clonal takeover of intestinal crypts fit well with a stochastic model, indicating that neutral drift was the driver of clonality in non-oncogenic situations (Snippert et al., 2010).

Another powerful tool in understanding competition and clonal dynamics is that of lineage tracing (Wagner and Klein, 2020). Somatic mutations have been used to map clonal contributions in the early embryo, creating a family tree of cells within our earliest stages of development (Park et al., 2021). The combination of mathematical modeling and a static, lentiviral barcode has also provided insight into clonal dynamics over the process of reprogramming, revealing competitive interactions (Shakiba et al., 2019). Additionally, as assays grow in complexity and adopt additional layers, cell types, and tissue architectures, single cell sequencing and lineage tracing will further enable us to understand the interplay between clonality and tissue identity, revealing hidden layers of homogeneity or heterogeneity that would otherwise be missed (Mckenna et al., 2016; He et al., 2021). One recent example looking at the haematopoietic compartment performed single cell RNA-seq with a modified pipeline, capturing both mtRNA and mRNA at single cell resolution from human samples (Miller et al., 2022). Using mtRNA to identify mutations, the authors were able to map the haematopoietic stem cell lineage. Using accompanying transcriptomic data, they correlated divergent differentiation trajectories with specific haematopoietic clones, connecting tissue-level function with clonal contributions to that function.

Finally, when looking to how the field of CC might aim to understand the flow of information from the cellular, to the tissue, and finally to the organism scale, key advances in molecular event recording technologies, which leverage the DNA as a data storage device, may bridge the gap. Early event recording technologies demonstrated the possibility for both recording and recovering cellular events in the history of individual cells from a single molecular input (Perli et al., 2016), with later works demonstrating recording systems that require two distinct signals to activate (Tang and Liu, 2018). As our knowledge of the molecular events and markers of competition grow, building synthetic genetic systems to record their presence and encode that information for recovery with single-cell resolution allows us to correlate molecular and cellular events with tissue-level outcomes through time (Sheth and Wang, 2018; Ishiguro et al., 2019). Critically, this removes the need to take snapshots of molecular events, instead allowing us to look back through the life-history of our model, identifying potentially complex events that have taken place days or weeks past.

To go beyond simple *in vitro* mixing assays, designing culture conditions that mimic the *in vivo* reality of multicellular systems would help bridge the gap. Advances in organoid tissue models have allowed for accurate representations of *in vivo* architecture while also incorporating cross-talk between multiple tissue types, striking a balance between biological reality and feasibility (Wimmer et al., 2019; Koike et al., 2021). With multi-organoid systems engineered from the ground up, we gain the ability to probe and control interactions between tissue types, capturing complexity that would otherwise be missed.

Synergy between the above tools provides a strategy to approach capturing information at the molecular, cellular, tissue, and organismal level. When conceptualizing an idealized competition experiment, the combination of competitive event recording and clonal lineage tracing in a full animal model would allow researchers to discover where these cellular battlefields lie, and which clones have shaped and overtaken their environment *via* these interactions. Coupling this molecular information with tissue function and organismal readouts would allow direct connection between molecular interactions and outcomes of the animal. Additionally, it would become possible to work backwards, performing insults or creating conditions that have been shown to affect organismal or tissue health and characterizing clonal or competitive interactions at the molecular level.

Ultimately, the field of cell competition is poised to mature from a novelty of mixing cells to a holistic process at the core of multicellularity, one that is a driving force in tissue homeostasis and function. Facilitating this transition are numerous advancements in adjacent fields, giving researchers the capability to ask deeper questions on the nature and evolutionary basis of the intriguing cellular behaviour that is CC.
